# Challenges for Researchers Investigating Contraceptive Use and Pregnancy Intentions of Young Women Living in Urban and Rural Areas of Australia: Face-to-Face Discussions to Increase Participation in a Web-Based Survey

**DOI:** 10.2196/jmir.2266

**Published:** 2013-01-21

**Authors:** Danielle L Herbert, Deborah Loxton, Deborah Bateson, Edith Weisberg, Jayne C Lucke

**Affiliations:** ^1^UQ Centre for Clinical ResearchUniversity of QueenslandBrisbaneAustralia; ^2^Research Centre for Gender, Health and AgeingUniversity of NewcastleNewcastleAustralia; ^3^Family Planning NSWSydneyAustralia

**Keywords:** Rural, urban, Australia, contraception, pregnancy, participant recruitment, women’s health, Facebook, social media, web-based survey

## Abstract

**Background:**

It is imperative to understand how to engage young women in research about issues that are important to them. There is limited reliable data on how young women access contraception in Australia especially in rural areas where services may be less available.

**Objective:**

This paper identifies the challenges involved in engaging young Australian women aged 18-23 years to participate in a web-based survey on contraception and pregnancy and ensure their ongoing commitment to follow-up web-based surveys.

**Methods:**

A group of young women, aged 18-23 years and living in urban and rural New South Wales, Australia, were recruited to participate in face-to-face discussions using several methods of recruitment: direct contact (face-to-face, telephone or email) and snowball sampling by potential participants inviting their friends. All discussions were transcribed verbatim and analyzed using thematic analysis.

**Results:**

Twenty young women participated (urban, n=10: mean age 21.6 years; rural, n=10: 20.0 years) and all used computers or smart phones to access the internet on a daily basis. All participants were concerned about the cost of internet access and utilized free access to social media on their mobile phones. Their willingness to participate in a web-based survey was dependent on incentives with a preference for small financial rewards. Most participants were concerned about their personal details and survey responses remaining confidential and secure. The most appropriate survey would take up to 15 minutes to complete, be a mix of short and long questions and eye-catching with bright colours. Questions on the sensitive topics of sexual activity, contraception and pregnancy were acceptable if they could respond with “I prefer not to answer”.

**Conclusions:**

There are demographic, participation and survey design challenges in engaging young women in a web-based survey. Based on our findings, future research efforts are needed to understand the full extent of the role social media and incentives play in the decision of young women to participate in web-based research.

## Introduction

Little information is available about how young women use and access contraception in any region of Australia, urban or rural. The need for information in this area is long overdue, as has been noted in the literature [1,2]. Existing Australian research into sexual activity, contraception, and pregnancy includes the National Survey of Australian Secondary Students from 1992 to 2008, prior to the students leaving school [3], and the Australian Study of Health and Relationships that surveyed adults aged 16-64 years [4,5]. Further research includes the Australian Longitudinal Study on Women’s Health (ALSWH) of the cohort born in 1973-78. They have been followed since 1996 at age 18-23 years [6]. Additional maternal health data from the birth of the first child to the end of women’s reproductive lives are reported annually in Australia [7]. However, there remains limited research specifically on the contraceptive use and pregnancy intentions of the new generation of young women.

It is difficult to capture the voice of young people living in rural areas in large population studies. For example, in 2010, New South Wales (NSW, the most populous state in Australia) had a population of 7.2 million, of which 14% were aged 15-24 years [8]. An analysis of population data highlighted demographic challenges as most young women in NSW live in major cities: only 16% of women aged 15-24 years lived in inner regional areas and 5% lived in outer regional and remote areas [8,9]. From age 20 years, the proportion of young women living outside the major metropolitan area of Sydney decreased across all other areas of NSW, indicating the movement of young women from rural areas to the greater Sydney region. Age-specific fertility rates in NSW are lower than the national rate in Australia, respectively, for 15-19 years (12.9 and 15.5 births per 1000 women) and 20-24 years (49.3 and 52.5 births per 1000 women) [7,8]. However, the fertility rates are dramatically higher in rural areas compared to rates in urban areas. While web-based recruitment can overcome any geographical limitations, response rates to population studies cannot be calculated using established methods because the number of potential participants is unknown [10].

There are many challenges in researching the health of young people, especially those living in rural areas [11]. Galea and Tracy (2007) have reported on the reasons for declining participation in epidemiologic studies and the resulting difficulty in recruiting population-representative cohorts: people are less likely to participate if they do not see the direct relevance to their personal lives. However, women who are employed and have a higher socioeconomic status are more likely to participate [12]. Differences in response rates in population-based studies may be accounted for by the sampling strategy used to identify eligible persons [12,13], including web-based recruitment through Facebook advertising [14]. A modern challenge for engaging young people in research is their preference for social media and the pursuit of a digital life [15]. An Australian survey of household Internet subscribers in 2010-2011 found social networking was one of the most popular Internet activities [16]. Almost all (96%) persons aged 18-24 years had access to the Internet and used their connection for emailing (93%) or social networking and online gaming (86%) [16]. A 2009 survey of 196,000 young people aged 15-24 years and living with a disability found 86% had access to the Internet at home [16]. Studies of the Internet activity of Australian households show that almost all households access the Internet either daily or weekly [16]. The use of social media by young Australian adults is similar to young American adults [17].

Little is known about how young women access contraception, particularly in rural areas of Australia. It is assumed that many young women visit their local GP for advice about contraception. In Australia, from 1998, standard general practice consultations have been monitored through the Bettering the Evaluation and Care of Health (BEACH) program [18]. Taylor et al (2010) analyzed BEACH data from 1994 to 2009 and found a reversal in the historical trend for extended consultations and the emerging preference for short appointments suited to single-issue consultations [19]. However, the comprehensive assessment of an individual’s need for contraception and pregnancy advice may require extended multi-issue consultations. Although a prescription to continue the oral contraceptive pill requires a short consultation, women who need to change their contraceptive method and be informed about new methods are likely to require an extended consultation or multiple visits.

Taylor et al (2010) recognized that a limitation of their study was the focus on individual services and not the assessment of individuals and their longitudinal use of services [19]. In 2001, adolescents aged 15-24 years had fewer visits to a doctor compared to older Australians, but most young women were attending for issues related to contraception or pregnancy [20]. Mazza et al (2012) used BEACH data to analyze contraceptive management by GPs and found a rate of 60 per 1000 consultations focused on contraception for women aged 12-54 years regardless of their residence in urban or rural Australia [21]. The extent to which young women get contraceptive information, advice, and services from locations other than their local GP is not known.

In order for research findings about how young women use and access contraception to inform policy and practice, it is important to engage young women in research design and implementation. This study aims to identify the challenges involved in engaging young women aged 18-23 years, especially those from rural areas, to participate in a web-based survey on contraception and pregnancy. Further, this study investigates the challenges involved in engaging young women to complete multiple follow-up web-based surveys in order to study changes over time in contraceptive use and pregnancy intentions.

## Methods

### Study Design

This study is part of a larger project called Contraceptive Use, Pregnancy Intention and Decisions (CUPID) of young Australian women. CUPID aims to shed light on the reasons for using, or not needing, contraceptive methods among young women, despite the apparent widespread availability of contraception. This study aims to identify how to encourage the participation of young women in a web-based survey, about contraception and pregnancy, through face-to-face discussions with young women. Ethical approvals were granted by committees at the University of Queensland, University of Newcastle, and Family Planning NSW. Additional information on CUPID is available from the project website.

### Sample and Recruitment

Young women aged 18-23 years and living in urban and rural NSW, Australia, were invited to participate in focus group discussions. Advertising for the discussions was via posters and information sheets displayed at the University of Newcastle, Family Planning NSW clinics (Ashfield, Fairfield, Penrith, Newcastle, Dubbo), educational settings (library), and one workplace setting with a trainee program including young women aged 18-23 years. Recruitment was achieved using direct contact (face-to-face, telephone, or email) and potential participants inviting their friends (ie, snowballing sampling). No financial incentives were given for participation in the study, but refreshments were provided during the discussions. Women who offered to participate but were aged outside the specified age range were excluded.

### Procedure

Ten semi-structured focus group discussions (lasting between 30 and 60 minutes) were conducted with participants from August to September 2011 by one member of the research team (DH) and an assistant (CR). The focus groups were held in urban areas at clinical locations in Sydney (Ashfield, Penrith) and Newcastle. In rural areas, the focus groups were held at clinical (Dubbo), educational (Tamworth), and workplace (Muswellbrook) locations local to the participants. Interviews were conducted with single participants where it was not possible to form a focus group of two or more participants aged 18-23 years, or the participant specifically requested to have a one-on-one discussion. The interview followed the focus group question schedule.

Before commencing the discussion, participants were given an information sheet describing the aims of the study, a consent form, and short survey to gather information on age, study or work patterns, and Internet use. All discussions were audio recorded after written consent was provided by the participants. A question schedule was used to stimulate discussion and included open-ended questions to encourage participants to give a broad range of responses. The interviewer (equally DH or CR) prompted the participants where appropriate to elicit more detailed information. Participants were asked to avoid revealing their personal experience of contraception and pregnancy but to focus on their reaction to survey questions on these topics. Other topics included access to the Internet, preference for using computers, smartphones or other devices, and responding to sensitive questions. The non-interviewing researcher (equally DH or CR) took notes throughout and summarized the discussion to the participants at the end of each discussion and invited corrections to the recording.

### Coding and Analysis

All discussions were de-identified, transcribed verbatim, and manually analyzed using thematic analysis. Our overarching aim was to identify important recommendations for research about contraception and unplanned pregnancy. We were interested in examining broad themes with which to frame recommendations, rather than detailed content analysis. We therefore employed a grounded theory approach [22] to examine the comments provided in the interview about participation challenges and survey challenges. Printed interview transcripts were read by DH. Statements that, directly or indirectly, referred to participation or survey challenges were identified. Excerpts were sorted into initial groupings by DH. These excerpts revealed five themes relating to participation challenges and seven themes relating to survey challenges. The research team reviewed these themes and agreed on the coding framework. All interviews were then coded by DH according to the identified themes using NVivo 9 (QSR International Pty Ltd., 2010). JL reviewed the transcripts and coding in detail and any minor discrepancies were resolved, and interpretations developed, with in-depth discussion to reach a consensus.

## Results

Twenty young women aged 18-23 years participated in the discussions (Table 1). Focus groups were conducted with five groups of participants at Dubbo (2 participants in a clinical setting), Newcastle (2 participants in a clinical setting), Sydney (one group of 2 participants and one group of 3 participants, both in a clinical setting,) and Muswellbrook (6 participants in a workplace setting). Due to recruitment difficulties or participant request, interviews were conducted with single participants at Tamworth (2 participants in an educational setting), Sydney (1 participant in a clinical setting), and Newcastle (2 participants in a clinical setting). Participants were living in urban (n=10) and rural (n=10) NSW; mean age 20.8 years. Half (n=10) of the participants were working only and not continuing with study after high school: most of these young women lived in rural areas (7/10). Participants from rural NSW were younger, working rather than studying, and unlikely to be attending university compared to participants from urban NSW. All participants used either computers or smartphones to access the Internet on a daily basis.

Two key domains were identified from the discussions: participation challenges to encourage young women to participate in a web-based survey and survey design challenges to ensure they would complete a survey including sensitive questions about sexual activity, contraceptive use, and pregnancy (Table 2). Direct quotes are included to provide contextual information for the identified domains.

**Table 1:** Characteristics of participants living in New South Wales (NSW, Australia).

Categories		Urban NSW	Rural NSW	Total
		n=10	n=10	n=20 (%)
**Age**				
	18-19	0	5	5 (25)
	20-21	6	2	8 (40)
	22-23	4	3	7 (35)
**Occupation**				
	Studying	6	1	7 (35)
	Working	3	7	10 (50)
	Studying & working	1	0	1 (5)
	Full-time mother	0	2	2 (10)
**Highest qualification**				
	Middle high school	0	2	2 (10)
	Senior high school	4	5	9 (45)
	Technical college	0	2	2 (10)
	University	6	1	7 (35)
**Internet use**				
	Daily	10	10	20 (100)
**Internet access**				
	Computer	3	4	7 (35)
	Smartphone	0	2	2 (10)
	Computer & smartphone	7	4	11 (55)

**Table 2:** Thematic analysis of focus group discussions (General = 19-20 participants, Typical = 10-18 participants, Variant = 1-9 participants).

Domains and subdomains		Themes	Frequency
**Participation challenges**			
	Internet access	Connect to Internet surveys	General
		Low coverage in some rural areas	
		Cost of access and download quota	
	Social media	Daily use of Facebook instead of email	General
		Free access to Facebook on mobiles	
	Incentives	Prefer financial incentive	General
		Want to know chance of winning prizes	
	Privacy	Confidentiality, trust, and security	Typical
		Reluctance to provide contact details	
	Prior knowledge	Want to know about the study before being asked to participate	Variant
**Survey challenges**			
	Format	Design of questions	Typical
		Mix of short and long questions	
		Up to 15 minutes to complete survey	
		Eye-catching and bright colors	
	Content	Sensitive questions okay if given option “prefer not to answer”	Typical
	Relationships	Range of potential relationship situations	Variant
		Committed versus casual relationships	
	Contraception	Some women use the term “protection”	Variant
		Never heard of some contraceptive methods	
	Pregnancy	Relationship status associated with pregnancy intentions	Variant
	Feedback	Want summary information of findings	Variant
		Like to compare themselves to the summary	
		Use of summary to inform their local area	
	Reminders	Continually receiving SMS reminders from range of companies	Variant
		Will tolerate 2-3 reminders to complete follow-up surveys but restrict to 1/week	
		Need a deadline to prompt survey completion	

### Participation Challenges

A recurring theme in the discussions was the ongoing cost of using the Internet, either on a smartphone, mobile phone or home computer: “But yes, being on the Internet and it’s going to cost you money ... because the Internet is pricey ... it’s just once you’re on there it’s like tick, tick, tick, money gone.” The constant awareness of the increasing Internet charges discouraged participation in a web-based survey. However, free access to social media through most mobile network service providers resulted in most participants using social media for almost all personal communications: “At the moment because I’m with [mobile network provider] ... you get free Facebook access, so that’s the only reason why I go on Facebook, because it’s free.” The majority of participants suggested the use of social media as the “only way” to engage their attention and raise awareness of the web-based survey.

An incentive to participate was desired by almost all participants: “I think it’d probably have to be like $20 [gift card] ... I do think that people do sometimes get a bit greedy, and it’s pretty sad that people just won’t do it to help people out.” Privacy and confidentiality were key concerns about participating in web-based surveys. Participants were concerned about providing their contact details especially if there would be future unexpected contact: “... and they want to interview me or, I don’t know, talk to me over the phone, then I wouldn’t necessarily give it.” Further, participants were worried about who wanted their information: “It’s not a freaky guy behind the computer just trying to get information about you”. Participants also wondered how their responses would be used: “Just you’re worried about where your information is going to go, and who’s going to get it.”

### Survey Design Challenges

Participants wanted the survey to be as short as possible (15 minute maximum) otherwise they were likely to leave the web-based survey and move to other websites. Asking sensitive questions was appropriate as long as “I prefer not to answer” was given as a response option:

You might still get people who are going to deny it when it’s true, because I’ve probably lied in questionnaires before about - oh sorry - but certain questions I was like, well, I don’t want to answer that. People would rather put a lie than leave it blank.

In the absence of this response option, most participants admitted they would give a false response in the survey.

Participants wanted to receive a combined feedback and reminder to complete the follow-up surveys because this information indicated their prior participation had contributed to some results: “… if you get some results back so you know... this is what your information gave, so then the next time they know it’s actually going to do something ... they’re more willing to do the survey in the next six months.” Participants from rural areas identified the benefit of using the feedback to advocate for change in their own community:

Or you did show a comparison because it’s rural and city. Show us the comparison. We’re rural, we want to see where we’re lacking and why we haven’t got the same things so that maybe, I don’t know how, but we could pitch it towards our community to try and get it going.

Many participants suggested certain personality types who were proactive, organized, and appreciated the benefits of research would always be more likely to participate in a web-based survey than other types of young women.

## Discussion

### Principal Findings

This study examined two key challenges to researchers in engaging young Australian women in studies of contraception and pregnancy. These areas are (1) ensuring adequate participation and (2) appropriate survey design to ensure the web-based survey is completed. Challenges relating to achieving adequate participation in a survey included the adequacy of participants’ access to the Internet, the use of social media, and avoidance of additional financial costs to access the web-based survey for young women living with socioeconomic disadvantages. Financial incentives were identified as the most effective method of ensuring recruitment of participants for short web-based surveys. Survey design challenges included the need for the survey to be short, to include the option to not answer questions, and for participants to receive feedback about the results. These challenges indicated that web-based surveys should be tailored to the needs of young women, especially those living in lower socioeconomic circumstances.

### Implications for Researchers

The web-based recruitment of research participants have been found to overcome the reluctance of young people to attend face-to-face discussions [23] and can reach hidden populations, eg, people with disabilities [10]. The use of the most popular social networking site, Facebook, has been identified as effective for web-based recruitment [14]. Fenner et al (2012) found that from 7940 Australians who clicked on the Facebook advertisement of their project, 3.5% went on to participate in the study. Among the young female participants, 28% were from a rural area, which was higher than the 21% of the target population from a rural area [14]. A US study of adult substance use also recruited through targeted Facebook advertising and successfully recruited 10% (n=1548) of the 14,808 people who clicked on the Facebook advertising, met the inclusion criteria and completed the survey [17]. However, web-based recruitment cannot occur when there is a digital divide beyond economics, eg, people living in rural Australia with unreliable or non-existent Internet availability. Notwithstanding these limitations, the promotion of research participation to young people as a means to have a voice, be heard, and contribute to their community may be the key driver to participate [24]. The findings of this study have shown that young women, especially those in rural areas, may be willing to participate in web-based research on issues important to them that will help them in their local community. Further, the clear message from young women was the need for a social media presence to grab their attention and ensure their participation.

One option that has been used to counteract the demographic challenge presented by the unequal numbers of young women in rural and urban areas of Australia is the use of a stratified random sampling frame to ensure young women living in rural areas are well represented and the findings are not urban-centric. Such a sampling frame is an established method, eg, the ALSWH has a successful history of recruiting and maintaining three generational cohorts of Australian women using mailed invitations and surveys [6,25]. However, the preference of young women to use social media or email instead of postal mail limits the effectiveness of using a similar methodology in a new generation of young women [14,15]. Participants in the ALSWH and in the Fenner et al (2012) study were broadly representative of the Australian female population with recognized over-representation of higher educated women whether the participants responded to postal invitations [6] or Facebook advertising [14]. A limitation of web-based recruitment, eg, social media, and subsequent volunteer participation is the potential loss of sample representativeness and generalizability of the findings. However, a very low response to postal invitations based on a stratified random sampling frame would also result in a non-representative sample of young women. A challenge for researchers is to adapt to generational changes within their target population and choose the least limiting web-based methodology while ensuring sufficient statistical power from their sample.

This study has identified key design features of a web-based survey to increase the likelihood of young women’s ongoing commitment to follow-up web-based surveys. Young women need to be reassured that their anonymity and confidentiality are maintained. They need to feel supported and secure that the researchers conducting the survey are legitimate and trustworthy by having recognizable logos and contact details. Young women agreed that university and government logos were seen as more legitimate than other novel logos viewed on websites, and these logos would encourage their participation. Feedback was imperative to ensure their continued commitment to the research. A study from the University of Wisconsin, USA, showed the suggestion of sending future email reminders to finish a partially completed survey was sufficient to encourage participants to immediately finalize the survey [26]. Further, the findings reported from this current study showed young women had a limited tolerance of reminders and preferred a short survey to be completed as quickly as possible.

This study successfully engaged a group of young women from rural and urban NSW in the development of a web-based survey of contraception and unplanned pregnancy. The findings of these discussions have implications for the research design and implementation of web-based surveys about sexual activity, contraception, and pregnancy, which must include potentially sensitive questions [27]. It is imperative to offer young women an appropriate range of response options to avoid forced responses that create false or blank reporting [27]. Reliable data are needed in order to make recommendations for policy and clinical practice interventions to reduce rates of unintended pregnancy.

### Conclusions

Information about contraception use and pregnancy intention in young Australian women is essential to inform policy but data are lacking, especially for those living in rural areas. Based on our findings, future research efforts are needed to understand the full extent of the role social media and incentives play in the decision of young women to participate in web-based research.

## Abbreviations

ALSWH: Australian Longitudinal Study on Women’s Health
BEACH: Bettering the Evaluation and Care of Health
CUPID: Contraceptive Use, Pregnancy Intention and Decisions of young Australian women
NSW: New South Wales, Australia
